# Steering the course: targeting and exploiting surface receptors in phage therapy

**DOI:** 10.1128/jb.00375-25

**Published:** 2025-12-08

**Authors:** Veronica N. Tran, Lori L. Burrows

**Affiliations:** 1 Department of Biochemistry and Biomedical Sciences, The Michael G. DeGroote Institute for Infection Disease Research, McMaster University 3710, Hamilton, Canada; Geisel School of Medicine at Dartmouth, Hanover, New Hampshire, USA

**Keywords:** cell surface, bacteriophage, *Pseudomonas aeruginosa*

## Abstract

The global rise of antibiotic resistance highlights the urgent need for alternative therapeutics such as bacteriophages (phages). Phages exert selective pressure that can “steer” bacteria toward reduced pathogenicity, increased antibiotic susceptibility, or immune clearance. A receptor-focused steering strategy is especially powerful since many phage receptors are also critical for bacterial fitness and virulence. Effective therapy requires identifying these receptors during characterization and combining phages that target distinct, conserved structures to minimize resistance. We review the current state of phage steering research and highlight guidelines emphasizing receptor identification for rational, durable therapeutic design. While this review focuses on *P. aeruginosa*, the findings and implications can be extended to other multi-drug resistant pathogens.

## PHAGE STEERING IN TREATMENT OF MULTIDRUG RESISTANT INFECTIONS

Antibiotic resistance (AMR) continues to be a global health issue, with morbidity and mortality rising each year (1). This situation emphasizes the need for new therapeutics and the reconsideration of bacteriophages (phages) for therapy. Phages are viruses that target only bacteria. Prior to the discovery of antibiotics, phages were used to treat bacterial infections, with the first reported case of phage therapy in 1921 (2). Once antibiotics were discovered, they became preferred treatments due to their broader host spectrum and lower risk of bio-contamination during production (e.g., endotoxin contamination). As a result, phage therapy became less popular, especially in Western countries (3). While therapeutic use of phages declined, their application as research tools has continued. Phage susceptibility can be used as a readout to evaluate if bacteria produce certain receptors (4, 5) or to assess receptor function (6). Phages are also used in lab-based diagnosis platforms to detect bacterial species of interest (7).

Since the early 2000s, there has been a resurgence of interest in phage therapy as a potential solution to the AMR crisis, as demonstrated by an increase in PubMed searches for relevant terms (8). This interest, along with technological advances that allow for better characterization and improved preparation of phages for therapy, has encouraged the renaissance of phage therapy for difficult-to-treat AMR cases. Bacteria can adapt to phage pressure and experience trade-offs between the development of phage resistance and loss of traits, such as virulence or fitness (9–11). The strategy of using carefully chosen phages to intentionally select for resistant strains that have lost specific traits is referred to as phage steering (12). Steering focuses on controlling the outcome and downstream effects of inevitable bacterial resistance, rather than simply eliminating bacteria through phage killing.

Phage receptors share many attributes with molecules used successfully as candidate vaccine antigens, as most are surface-exposed structures at the interface between bacteria and the environment, contributing to fitness, virulence, and antibiotic susceptibility. Bacteria can become resistant to phages through changes in receptor function and/or expression, reducing infectivity or persistence (13–15). Here, we review the spectrum of phage receptors, their role in phage infection, bacterial pathogenicity, and relevance in initial phage selection. We also propose a framework for consideration of phage receptors in phage research and therapy. This review focuses mainly on *Pseudomonas aeruginosa*—a Gram-negative, opportunistic pathogen associated with nosocomial infections in immune-compromised patients, including those with cystic fibrosis (CF), burn wounds, sepsis, post-surgical infections, ventilator-associated pneumonia, and urinary tract infections (UTIs) (16–19). This multi-drug-resistant (MDR) pathogen urgently requires new therapeutics, as indicated by its place near the top of the WHO priority pathogen list and as an ESKAPE pathogen (20, 21). There are many examples of *in vivo* preclinical models and individual patients infected with MDR *P. aeruginosa* that have been treated successfully with phage therapy (22–27), and the first-ever randomized controlled trial of phage therapy (PhagoBurn) was designed to evaluate treatment of *P. aeruginosa-*infected burn wounds (28). Experience and lessons learned from the growing field of *P. aeruginosa* phage therapy are useful for the design of similar treatments for other MDR pathogens.

## PHAGE REPLICATION CYCLES

Most phages use one of two replication cycles—lytic or lysogenic. Strictly lytic phages infect, replicate, and release phage progeny by bursting or lysing cells, leading to host cell death (29). In contrast, lysogenic phages replicate alongside their host bacterium by integrating their genetic material into the host genome (prophage) (30) or as a plasmid (31). Bacteria containing a prophage are referred to as lysogens. In response to bacterial stress (i.e., DNA damage, toxins, phage superinfection), lysogenic phages may switch to the lytic cycle, thus inducing cell death (32–34). This switch can also occur randomly (spontaneous induction) (35). Some prophages may release progeny independent of cell lysis (36, 37). Prophages can express genes that modulate cellular processes or alter the expression of their original receptor to prevent infection by related phages (superinfection immunity or exclusion) (32, 38, 39). They may also encode antibiotic resistance genes or factors that alter fitness and virulence of the lysogen (39, 40). Lytic phages are favored for therapy because of their inability to integrate into the genome, since the goal is to eliminate the pathogen.

If phages bind successfully to their host to initiate infection, defense mechanisms that recognize phage nucleic acids and interfere with phage replication may become activated. Restriction modification systems recognize foreign nucleic acids based on sequence and/or methylation patterns and cleave them (41). CRISPR-Cas systems act as bacterial immune factors, facilitating the recognition and cleavage of phage nucleic acid sequences to which the cell has previously been exposed (42). If phages evade these mechanisms, bacteria may enact abortive infection (Abi) strategies. There are many different Abi mechanisms; all generally deplete critical cellular resources or halt cell metabolic and replication processes to prevent phage replication, resulting in programmed cell death that prevents propagation of the phage progeny and infection of kin cells (43). The list of phage defense mechanisms continues to grow rapidly as new ones are identified. A comprehensive list is beyond the scope of this review, but for further exploration, readers are directed to these excellent sources (44–48).

Despite this growing array of defense mechanisms, a recent study suggested that bacterial surface components are more important determinants of successful phage infection than subsequent anti-phage strategies (49). Therefore, defining and characterizing phage receptors is a logical approach to ensure maximum efficacy of phage therapy and phage steering.

## PHAGE RECEPTORS: WHAT ARE THEY AND WHY SHOULD THEY BE TARGETED FOR STEERING?

Phage adsorption to a receptor is the essential first step in phage infection. This is considered to be a random event, as phages are not motile and their passive transport is influenced by their environment (50). Depending on the type of phage, the mechanisms for binding will vary. Generally, surface-exposed binding proteins on the phage interact first reversibly and then irreversibly to cell surface receptor(s) (51, 52). Tailed phages often have receptor binding proteins located at the tips of their tails, referred to as tail fibers, tail spikes, or tail tips (51). Some phages can encode multiple types of tail fibers, allowing for binding to more than one receptor (53). Filamentous phages, such as *P. aeruginosa* phage Pf, interact with their receptors using one end of the phage body (54). For most phages, receptor binding will trigger conformational changes in the capsid that lead to injection of genetic material, initiating phage replication in the target cell (51, 52).

### Qualities of an ideal phage receptor

**Fig 1:**
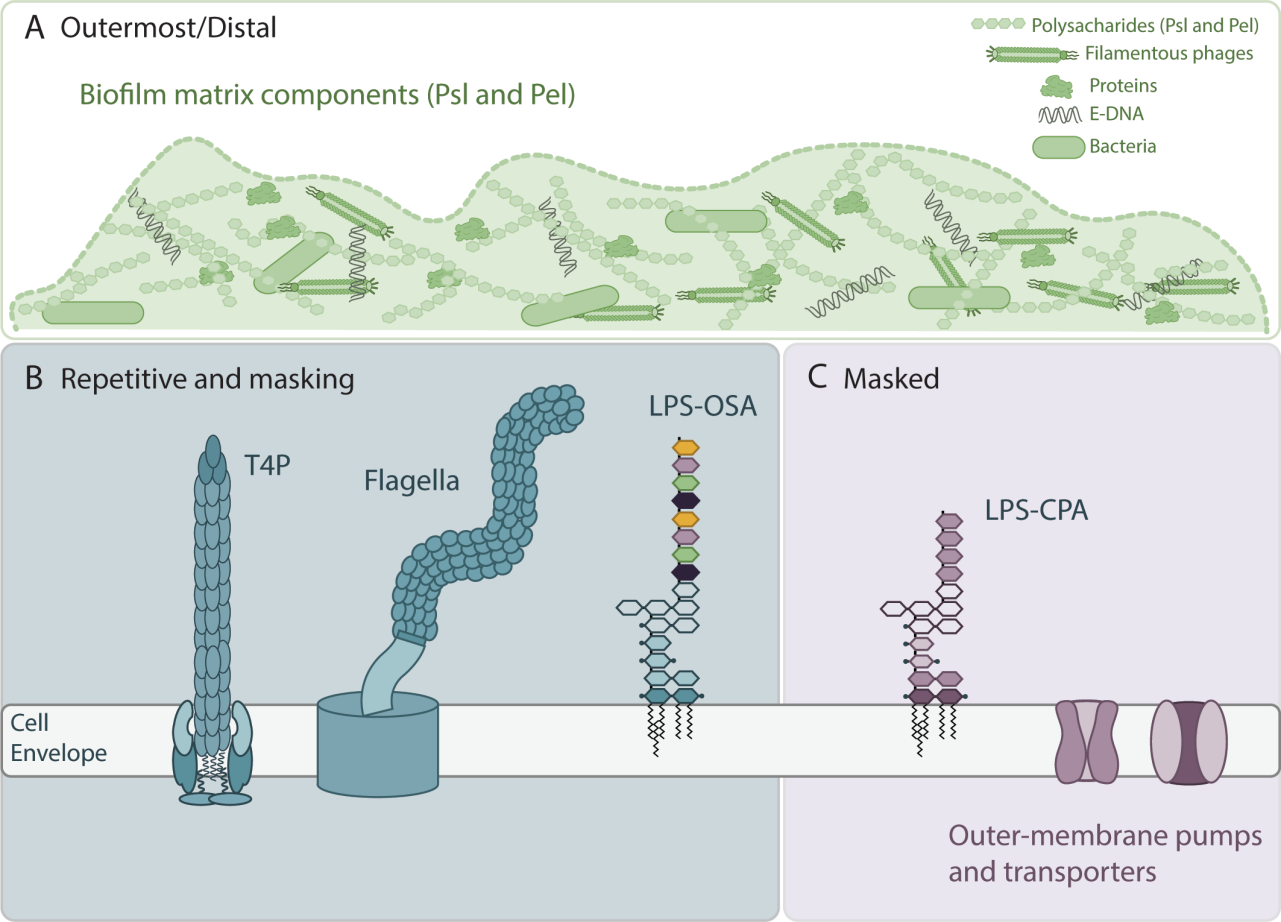
Candidate receptors to target for phage steering. Biofilm matrix components, such as Pel and Psl polysaccharides, (**A**) are the outermost/distal candidate receptors and may be targeted first. This can allow for increased phage accessibility to the canonical receptors (**B**), such as type IV pili (T4P), flagella, and the O-specific antigen (OSA) component of lipopolysaccharides (LPS), composed of repeating units that could enhance binding avidity. Targeting these may then allow further access to receptors predicted to be masked by the canonical receptors (**C**), including the shorter type of LPS in *P. aeruginosa*, the common polysaccharide antigen (CPA), and outer membrane porins or nutrient receptors.

Since phage–receptor interactions are stochastic, phage receptors are often repetitive surface-exposed structures that ensure high likelihood of interaction. For example, most Gram-negative receptors are cell surface–associated or outer membrane structures composed of repeating sugar units, such as those found in biofilm matrix polysaccharides (Fig. 1A), lipopolysaccharides (LPS) (Fig. 1B), or protein filaments assembled from repeating protein subunits, such as flagellins or pilins (Fig. 1B) (55, 56). Biofilm matrices can also contain eDNA, proteins, lipids, and filamentous phages (Fig. 1A). For *P. aeruginosa*-specific phages, type IV pili (T4P), LPS, and flagella are the most frequently identified phage receptors (Fig. 1B) (14, 56, 57). These structures contribute to bacterial fitness and virulence in distinct ways and are therefore desirable phage steering targets. It is important to note that there can be considerable variation in T4P, LPS, and flagella subunits expressed by the target strains and/or species; in particular, pathogenic or transmissible strains may have specific surface markers. Receptor variation may have been shaped by historical exposure to phages that recognize those surface structures, selecting for mutations or structural modifications that allowed for escape.

T4P are protein nanomachines found in many species of bacteria (58). In addition to phage binding, these surface-exposed filaments mediate surface sensing (59), adhesion (60), and motility (61). These functions all contribute to the ability of the bacteria to establish infection through promoting biofilm formation and upregulation of virulence factors (60, 62). *P. aeruginosa* T4P are composed of major pilin subunits, classified into five different pilin groups (I–V) based on sequence and structural diversity (63), plus one of two sets of minor pilins and an adhesin called PilY1 that together form the pilus tip (64). Each *P. aeruginosa* strain expresses only one major pilin variant and one set of minor pilins. While the N-terminal ~30 residues of major pilins are nearly identical among different pilin groups, the remainder of the subunits have limited sequence identity. For example, the major pilins of well-studied strains PAO1 (group II) and PA14 (group III) are 28% identical overall, but only 18% identical when the highly conserved N-terminus segment is omitted. The variable portion forms the exterior surface of the pilus filament that is recognized by phages. Major pilins are the most common receptors, but phages that bind minor pilins have also been identified (65).

LPS forms the outer leaflet of the outer membrane and contributes to membrane integrity, stabilization of outer membrane proteins, and decreased permeability to exogenous compounds (66, 67). It is also broadly recognized by host immune systems (68). LPS consists of lipid A, with its membrane-embedded acyl chains, inner and outer core sugars, and long-chain O-antigen attached to a subset of those cores (Fig. 1B). *P. aeruginosa* is unusual among Gram negatives in that it constitutively co-expresses two chemically distinct O-antigens: the common polysaccharide antigen (CPA, formerly A-band LPS), and O-specific antigen (OSA, formerly B-band LPS) (69). CPA is a homopolymer of D-rhamnose, whereas OSA is a heteropolymer whose sugar composition, glycosidic linkages, and length depend on the specific serotype. Based on the International Antigenic Typing Scheme (IATS) classification, there are 20 different *P. aeruginosa* serotypes (69). More virulent and transmissible strains are associated with certain serotypes (70). It is interesting to note that even though CPA is highly conserved among *P. aeruginosa* strains, most known *P. aeruginosa* LPS-targeting phages recognize OSA. The specific role of different components of LPS in immune recognition varies between organisms, but the inner and outer core regions are better recognized by immune factors (67). Phage selection for resistant mutants with shorter or no O-antigen can increase immunogenicity and decrease bacterial fitness (71). O-antigens can also contribute to immune clearance, either through complement activation or cell death mediated by NETosis—neutrophil release of extracellular traps (ETs) containing bactericidal molecules (67, 72, 73). Thus, loss of O-antigens may favor alternate immune pathways.

OSA is on average longer than CPA (Fig. 1C) and may reduce CPA’s accessibility to phages (74–76). Alternatively, CPA, a homopolymer of the neutral sugar D-rhamnose, may be difficult for phages to bind with high affinity. The scarcity of phages that target CPA may be advantageous for *P. aeruginosa*, as CPA may continue to mask other potential receptors if OSA is lost in response to phage pressure. Phages that could use the highly conserved CPA as a receptor would be useful for therapy. Other potentially masked components include outer membrane components of efflux pumps, as well as common surface-associated proteins such as porins and nutrient transporters (e.g., TonB-dependent transporters) (77) (Fig. 1C). For example, OMKO1 is a *P. aeruginosa* phage thought to target the outer membrane component of a drug efflux pump (12). Loss of efflux pumps in response to phage pressure has the potential to resensitize drug-resistant isolates to antibiotics.

Flagella are key motility organelles found in many bacterial species (78). These whip-like filaments contribute to bacterial adhesion (79), upregulation of other virulence factors through activation of surface sensing pathways (80), and biofilm formation in *P. aeruginosa* (81). Like LPS, flagella are also broadly recognized by the host immune system to initiate immune response (82). *P. aeruginosa* flagellar filaments are composed of a-type or b-type flagellin subunits (83). a-type flagellins can be further categorized into subtype A1 and A2, while b-type flagellins are conserved (83–85). Knowing the specific receptor alleles expressed by a particular strain is important for the selection of appropriate phages, since they may not recognize all versions of a particular surface structure (15, 39, 86). Flagellum-targeting phages are less common than those that recognize T4P or LPS, although some have been identified (14). Phages may be less likely to encounter and adhere to the rapidly moving flagellum of a bacterium unless the bacteria are in environments where flagellar movement is restricted (87, 88). Flagella are longer than T4P and do not retract. Therefore, phages may not be able to reach the cell surface as easily to initiate infection once bound to flagella.

### Phage isolation sources and techniques can bias which receptors are identified

Most *Pseudomonas* phages described in the literature were isolated from wastewater or environmental sources, and their receptor profiles were established through selection of phage-resistant isolates of strains grown in laboratory media, which is optimized for rapid bacterial growth. These conditions are different from host environments and therefore may select for phages that use receptors that might have different patterns of expression or relevance *in vivo*. Continued use of traditional methods of discovering and characterizing phages likely contributes to the rediscovery of phages that target the same limited set of receptors including T4P and LPS. This problem is similar to the issue of antibiotic dereplication faced by groups searching for novel classes of antibiotics, where established screening methods lead to the frequent rediscovery of well-characterized compounds (89). Therefore, novel approaches and tools must be used in order to find phages that target non-canonical receptors.

Using defined bacterial mutants that lack these commonly recognized phage receptors during phage isolation represents a straightforward approach to look for phages with more diverse targets. For example, a Psl exopolysaccharide-targeting phage was recently identified using a *P. aeruginosa* strain devoid of the canonical receptors pili and flagella (90). Psl is a conserved component of *P. aeruginosa* biofilm matrices and is important for surface attachment and biofilm structural integrity (91–93). Since exopolysaccharides can provide phage protection, discovery of Psl targeting phages will lead to new ways to breach this barrier (90, 94). Known *P. aeruginosa* phage receptors are summarized in Table 1.

**TABLE 1:** *P. aeruginosa* phage receptors

Receptor	Function in the bacterial host	Example phage (reference)
LPS O-antigen (OSA)	Outer membrane integrity, immune recognition	D3 (86)
T4P	Motility, surface adherence, biofilm formation, cell signaling	PO4 (95)
Flagella	Motility, surface adherence, immune recognition	fMGyn-Pae01 (14)
Psl	Biofilm exopolysaccharide component	CLEW-1 (90)
OprM	Efflux pump	OMKO1 (96)

## BACTERIA MODIFY RECEPTORS TO ESCAPE PHAGE PRESSURE

**Fig 2:**
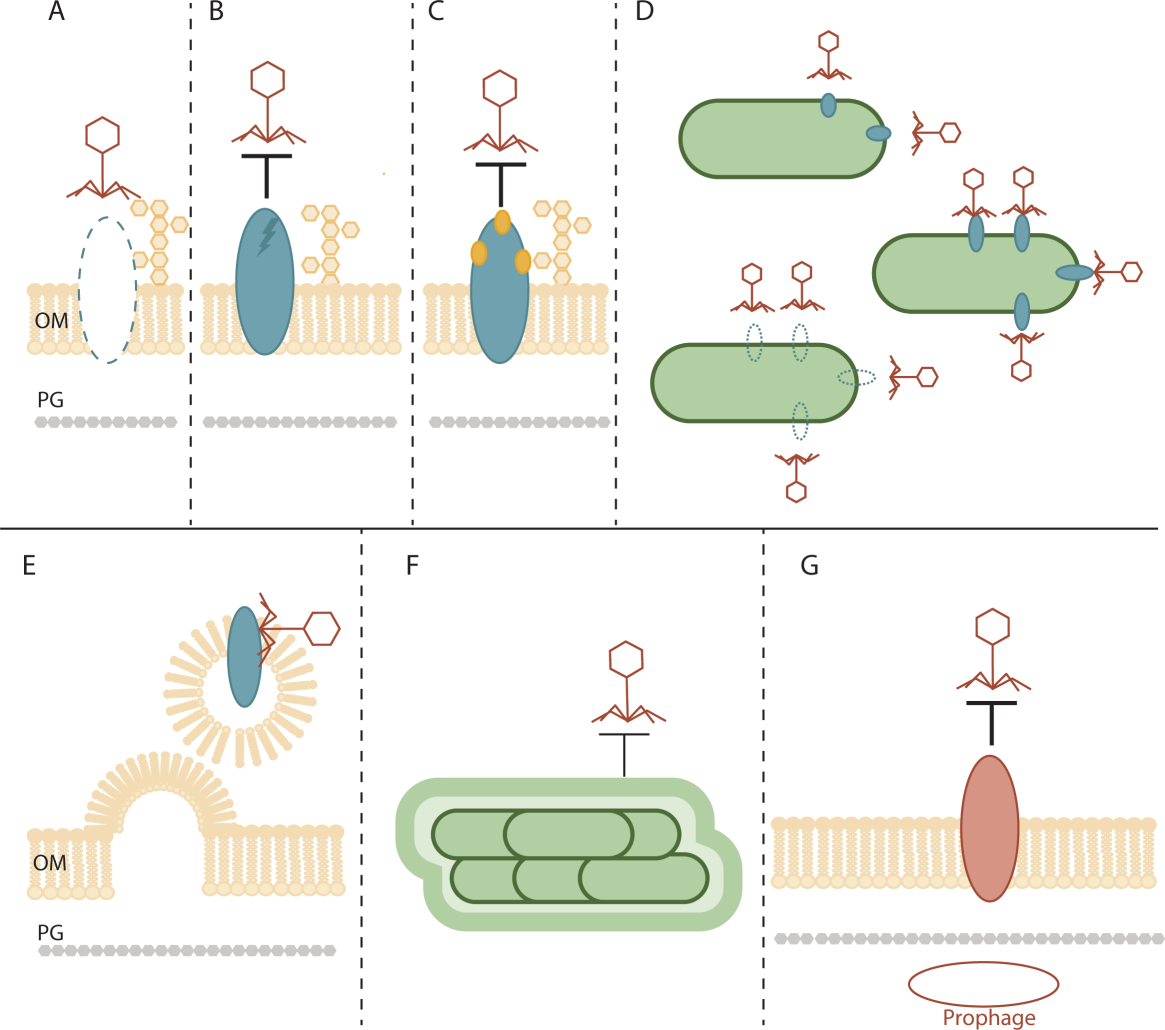
Receptor-based phage defense mechanisms used by *P. aeruginosa*. Single-cell level resistance mechanisms include (**A**) complete loss of the receptor, (**B**) modification of the receptor through mutations, or (**C**) post-translational modifications to block the receptor. Community-level resistance can be achieved through (**D**) phase variation of receptor expression, (**E**) release of outer membrane vesicles that contain the receptor, and (**F**) formation of extracellular matrices. (**G**) Lysogens can become resistant through expression of a prophage-encoded version of the receptor.

Due to the phage-bacterial arms race, phages must continuously evolve new strategies to evade bacterial defense mechanisms that target different steps of phage infection. Since the initial step of infection involves recognition of and binding to extracellular phage receptor(s) (51), bacteria have multiple ways to block this interaction, precluding the need for more elaborate strategies to suppress phage replication once the cell is infected. The most straightforward strategies are loss (Fig. 2A) or modification (Fig. 2B) of the receptor, through deletions or mutations in the receptor itself or in genes that are part of receptor biosynthetic pathways. Resistance through loss of receptors can be achieved through modest genetic changes. For example, single amino acid mutations in the pilus extension ATPase PilB conferred resistance to T4P-targeting phages by abolishing pilus assembly (97). Similarly, single amino acid mutations in glycosyltransferases involved in LPS biosynthesis provided protection against LPS-targeting phages by preventing addition of the O-antigen receptor (97). Mutations in *P. aeruginosa* resistant to the flagellum-targeting phage fMGyn-Paeo1 mapped to three different genes: *fliE*, encoding the hook; *fliC*, encoding flagellin; and *flgK*, which encodes the adaptor between the hook and filament (14, 98). All these mutations resulted in the loss of the flagellum filament and, consequently, the phage receptor. The loss of receptor expression or function as a result of small genetic changes, such as single-nucleotide-induced frameshifts, implies that reversing such phenotypes may also require only additional small changes that restore wild-type sequence. The propensity for phage-resistant mutants to revert to susceptibility following the removal of phage pressure has not been systematically evaluated but has important implications for resolution of infection following phage therapy.

Since loss of a receptor can have repercussions for bacterial fitness, phage receptors can also be post-translationally modified to block phages from recognizing them (Fig. 2C). *P. aeruginosa* strains expressing group I or group IV pili (e.g., strains PA1244 and Pa5196, respectively) glycosylate the major pilin PilA, blocking phage infection (15). While some *P. aeruginosa* flagellins are glycosylated (99, 100), the role of this modification in phage infection is not well understood. In other species such as *Campylobacter jejuni*, flagellar glycosylation is linked to phage infection, and this modification is predicted to evolve in response to phage pressure (101). Other post-translational modifications have also been reported. For example, phosphorylation or modification of pilins with phosphocholine (PC), phosphoethanolamine (PE), or phosphoglycerol (PG) has been reported in certain *Neisseria* species, but such modifications have not been reported in *P. aeruginosa,* nor is their potential impact on phage recognition known (102–104).

### Community-level strategies for phage resistance

There are also receptor-based strategies that mediate resistance at the community level. In response to particular environmental conditions or stressors, bacteria may undergo phase variation—a reversible change in gene expression facilitated by genomic or epigenetic variations (105). T4P and flagellum expression can be subject to phase variation and, therefore, reduce phage infection through changes in receptor availability (Fig. 2D) (105, 106). Since phase variation occurs stochastically, it may result in a mixture of subpopulations that express or do not express the receptor (107). This bet-hedging strategy promotes survival once phage pressure is removed (108). It is important to note that T4P and flagellum phase variation has not been reported in *P. aeruginosa*.

Bacteria can release outer membrane vesicles (OMV) that contain surface-exposed phage receptors (Fig. 2E). OMVs act as decoys and sequester phages away from the target cell, thereby reducing infection (109, 110). Biofilm extracellular matrices and the components (e.g., polysaccharides, proteins, extracellular DNA, filamentous phages) also protect bacteria from phage infection by acting as a physical barrier that limits phage transport toward the cells and masks other cell-surface phage receptors (Fig. 2F) (111–114). *P. aeruginosa* is a prolific biofilm producer both *in vitro* and *in vivo*, making this form of phage protection clinically significant. This physical type of defense can be countered by phages that express glycosyl hydrolases on their tails, allowing them to degrade polysaccharides and gain access to secondary cell surface receptors (115, 116). In *E. coli*, curli fibers, which are comprised of amyloid oligomers, confer phage protection by similarly blocking phage receptor access (117). Though a potential role in phage protection is unclear, amyloid proteins are also encoded by *P. aeruginosa* and confer protection against the predatory bacterium *Bdellovibrio bacteriovorus* (117).

### Control of host receptor availability by resident prophages

Receptor availability can be controlled or modified in lysogens via expression of prophage-encoded peptides or proteins (Fig. 2G). Most *P. aeruginosa* strains contain at least one prophage (118–122). They can prevent co-infection by related phages through superinfection immunity and/or exclusion mechanisms that can downregulate or modify the phage receptor to prevent phage recognition. For example, Tip and Aqs1 are related small proteins encoded by two different *P. aeruginosa* targeting phages (D31123 and DMS3) that reduce pilus extension by inhibiting function of the extension ATPase PilB (38, 123). As a result, lysogens expressing these respective proteins/peptides have little to no pilus expression, preventing infection by other T4P-targeting phages.

Prophages may also encode proteins or peptides that alter receptor composition without altering function. For example, phage JBD68 lysogens express a phage-encoded minor pilin (P-FimU) that is incorporated into the T4P in place of the host-encoded version, likely by outcompeting bacterial FimU in abundance (65). Since JBD68 is a T4P-targeting phage predicted to interact with the lower abundance minor pilins located at the tip of pilus, expression of this modified T4P component is thought to prevent subsequent infection of the lysogen by similar phages while maintaining pilus assembly and, consequently, host fitness. Similarly, lysogens of LPS-targeting *P. aeruginosa* phage D3 have a modified O-antigen structure compared to non-lysogens, changing their serotype from O5 to O16. D3 encodes an α-polymerase inhibitor that blocks the bacterial O-antigen polymerase (Wzy), enabling the phage-encoded β-polymerase (Wzy_β_) to shift O-antigen linkages from α1-4 to β1-4 (86). It also encodes Oac, which is responsible for *O-*acetylation of the O-antigen sugar, FucNAc . As a result, lysogens are protected from infection by other phages that recognize serotype O5 LPS, which serves as the receptor for D3.

## SELECTION OF PHAGE RECEPTORS BASED ON CONTEXT AND INFECTION TYPE

Phage infection can be proportional to the number of accessible receptors (124), and limited receptor availability may decrease therapy efficacy. Thus, when selecting which phage receptors to target for therapy, it is necessary to consider if the receptor is likely to be expressed and accessible *in vivo. P. aeruginosa* can be present in multiple states in infection, ranging from single cells to communities of biofilms in chronic infections. The effect of these varying phenotypes on phage receptor availability must be accounted for. Canonical receptors, such as T4P, LPS, and flagella, can be downregulated in biofilms (125, 126), and biofilm matrix components can prevent phages from accessing their target. *P. aeruginosa* biofilm infections are common among people with CF. Due to the unique environment of the CF lung, these infections can be nearly impossible to clear. CF lung physiology is abnormal (changes in cell types, epithelial structures, pH), and lungs express increased levels of mucin, in which bacteria become embedded in (127). These contribute to conditions favorable to the establishment of infection and reduced clearance of pathogens (128, 129). Downregulation of flagellin and pilin expression is seen in chronic CF-associated *P. aeruginosa* lung infections, and these conditions may affect the success of pilus and flagellum-targeting phage therapy.

Another consideration is the variation in LPS serotypes among isolates. LPS-targeting phages are often serotype-specific, and as in the case of D3, above, *P. aeruginosa* strains may undergo serotype conversion due to products of genes carried by prophages (86, 130). While a *P. aeruginosa* phage that can bind to LPS of different serotypes has yet to be reported, *Escherichia coli* LPS-targeting phages CBA120 and EP75 encode three and four, respectively, distinct tail spike proteins that facilitate their ability to recognize multiple serotypes (53, 131). However, since there are over 180 *E. coli* O-antigen serotypes, this “broad” host range is still quite limited (132). It is important to consider the serotype specificity of candidate LPS phages and to account for conversion events that may impact treatment efficacy.

Receptor expression at different replication and/or lifecycle stages should also be considered. For example, phages that target components of biofilms or that disrupt biofilms could be combined with T4P- and LPS-targeting phages. Matrix-targeting phages can promote dispersal of the biofilm and transition to the planktonic state, followed by re-establishment of microcolonies. T4P are upregulated upon surface contact, and therefore these colonies may be more susceptible to T4P-targeting phages (62). Biofilm disruption would also have the added benefit of increasing antibiotic susceptibility.

*P. aeruginosa* is often associated with nosocomial infections. Compared to environmental strains, hospital-associated strains of *P. aeruginosa* can be more resistant to antibiotics (133). Bacteria evolve quickly in response to antibiotic administration, sometimes creating distinct genetic clusters (134–136). There may also be differences between hospitals in terms of prevalent circulating strains. These factors may result in variations in phage receptor profile that reduce efficacy of phage therapy. The addition of exogenous therapeutic phages or antibiotics could induce lytic replication of resident prophages through activation of host stress pathways (137–139). These unintended additions to therapeutic phage cocktails might alter the expected trajectory or outcome.

Polymicrobial infections are common, especially in immunocompromised patients (140, 141). When patients are treated with phages that target only a subset of pathogens in these polymicrobial infections, community composition may shift to favor proliferation of another species. This was the case when an infected patient was treated with only *P. aeruginosa* phages. Although *P. aeruginosa* was cleared, infection with *Klebsiella pneumoniae* subsequently emerged (142). Phage therapy may also promote *P. aeruginosa* infection, as seen in a patient that was only treated with *Staphylococcus aureus-*targeting phages and had recoverable *P. aeruginosa* following treatment (142). In these cases, using a polymicrobial-targeting phage cocktail, which contains phages that target different species (143), is key to ensure total eradication of bacteria.

In immunocompromised individuals with chronic biofilm infections (144–148), phage steering toward immune clearance may not be an ideal approach, as the patients cannot efficiently clear phage-resistant bacteria. In these cases, steering toward increased antibiotic susceptibility may be the more useful approach. Bacteria in biofilm microcolonies are more resistant to antibiotics (149); therefore, disruption of the biofilm may be required for increased antibiotic sensitivity and infection resolution. In these cases, phage therapy may act as an aide, with the focus on antibiotics and appropriate adjuvants, when required, to achieve bacterial clearance. This will require specific examination of phage-antibiotic synergy in preclinical testing.

As with any form of therapy, unintended consequences may arise. Because phages will select for loss of their receptor, other phages that use the same target will be unable to infect resistant colonies. This issue emphasizes the importance of using phage cocktails or sequential administration of phages with different targets. Ideally, phage cocktails should include at least two phages that target different receptors to reduce cross-resistance and redundancy. Since most well-characterized *P. aeruginosa* phages target the T4P or LPS, a combination of phages that use these two receptors is a popular approach (150, 151). While this may be strategic, the high proportion of phages that use these receptors could mean that bacteria have developed more anti-phage strategies for these structures, thereby decreasing efficacy. Therefore, it is important to continue to search for phages that use other targets, including those that are normally masked or poorly expressed in laboratory conditions. Phages that target alternative receptors could be combined with phages targeting the typical receptors to reduce the development of resistance.

## CURRENT APPROACHES TO PHAGE THERAPY AND PHAGE STEERING

Phage therapy regulation falls broadly within the following classes: magistral use, where pre-approved phages are combined and administered by pharmacists on a case-by-case basis (e.g., Belgium) (152, 153); compassionate use, where each case requires approval by a central governing agency (e.g., Canada, USA, UK); or clinical trials, where the efficacy of a defined phage cocktail or treatment regimen is assessed in multiple patients (28, 154).

Phage therapy is considered safe and effective, with few adverse events (155). This may be attributed to phage specificity, as their propensity to target single species or strains reduces the risk of disrupting the patients’ microbiomes. Phages can be used in a broad range of indications. For *P. aeruginosa*, post-surgical (joint replacement, prostheses, transplants) infections that fail to respond to conventional antibiotics are candidates for phage therapy (142, 156, 157). Other common indications include burn wounds, as well as urinary tract, bone-related, and lung infections, including chronic infections of CF patients (142, 156, 157). Like antibiotics, phages can be administered via different routes, including intravenously, topically, by inhalation, or directly to the site of infection (142, 157). Most reported cases used multiple phages, creating a cocktail unique to each person (142, 157).

Pre-defined phage cocktails—approved combinations of phages that target single or multiple organisms and prepared by industry or health and research groups—have also been used (142). Two commonly used pre-established phage cocktails, BFC1 and BFC2, are prepared by the Queen Astrid Military Hospital (QAMH) in Belgium and used for treatment of *P. aeruginosa* polymicrobial infections (142, 158, 159). In some countries (especially Georgia and Russia), there is a long-standing practice of using less well-defined phage cocktails that are broadly available in different formats (oral, topical, suppository). They are prescribed for a number of common ailments based on symptoms or site, rather than on identification of a particular pathogen (160). These formulations are available in other countries through naturopathic practitioners and regulated as health supplements.

Because of standard-of-care expectations, lack of clinical trial data, and regulatory hurdles, phage therapy is still considered a last-resort treatment, used only when patients no longer respond to antibiotics due to resistance or adverse side effects. Typically, bacterial isolates will be retrieved from the patient and tested against a panel of phages for susceptibility. Phages are prepared according to local regulatory guidelines which may include microscopy, genomic sequencing, good manufacturing practices (GMP), and testing for endotoxins, contaminants, and titer (161). Depending on the indication and/or geographical location of the patient, therapeutic phages may be readily available. If not, unique phage preparations must be created for each patient.

The process of characterizing a new therapeutic phage can be lengthy. According to a retrospective analysis of the first 10 cases of phage therapy in the USA, the time between treatment request to administration varied from 28 to 386 days (median of 170.5 days) (162), making it a challenge for urgent cases. The first 100 cases in Belgium using the magisterial approach reported an average of 3 weeks, showing that having sets of diverse phages that are preapproved for safety can reduce the time to treatment (142). Despite its promise, phage therapy is not always available for patients. Only 5.8% (*n* = 260) of phage therapy requests submitted to the QAMH over a five-year period resulted in treatment, and about half were rejected due to lack of phages targeting the relevant bacterial species (142). The time constraints may be alleviated by improved characterization of phages prior to administration, inclusion in accessible phage libraries, and expansion of the host range of phages in such libraries. A grassroots effort by phage researchers Jessica Sacher and Jan Zheng, called Phage Directory (https://phage.directory/), attempts to connect researchers and clinicians around the world using email alerts to send out urgent requests for phages to treat challenging cases.

Most current forms of phage therapy use strictly lytic phages. However, recent *in vitro* studies showed that combinations of lysogenic phages plus antibiotics can eradicate bacteria, even when neither was successful alone, suggesting therapeutic potential (163–165). More studies are required to evaluate safety, especially since many lysogenic phages contain genes of unknown function whose impact on host biology is hard to predict. In the meantime, if lysogenic phages are the only available option to treat a particular patient isolate, they can be genetically engineered to render them unable to integrate into the genome. This strategy was used to successfully treat a CF patient with a drug-resistant *Mycobacterium abscessus* infection (166). The capacity to use lysogenic phages can expand both the number of phages available and, potentially, the receptor profiles.

### Examples of phage steering *in vitro*

*P. aeruginosa* antibiotic resistance continues to escalate, inspiring *in vitro* studies that focus on steering antibiotic-resistant strains of *P. aeruginosa* back to susceptibility. Isolates resistant to phage OMKO1 were reported to be more susceptible to antibiotics than parental strains (12, 96). Bacteria resistant to T4P- or LPS-targeting phages have reduced motility, biofilm formation, and sometimes growth rates, depending on the nature of the mutation (167, 168). Combined mutations in both pathways can cause even greater fitness deficits (169). Using LPS-targeting phages may contribute to increased antibiotic susceptibility due to selection for phage-resistant mutations that compromise outer membrane integrity, but directly targeting efflux pumps using phages like OMKO1 may have a more pronounced impact on minimal inhibitory concentrations (167, 168, 170).

### *In vivo* research on phage steering

The added impact and complexity of host immune systems on infection dynamics and phage therapy outcomes can be more accurately captured using *in vivo* models. When *Galleria mellonella* larvae were inoculated with strains resistant to LPS- or T4P-targeting phages, or both, only one LPS phage-resistant isolate showed reduced virulence, whereas more than half of the tested T4P phage-resistant, as well as 2- and 3-phage cocktail-resistant isolates, had reduced virulence (171). Thus, the ability to steer bacteria towards desirable clinical outcomes, such as immune clearance and reduced virulence, is achievable *in vivo*.

In Western countries, phage therapy is typically used in combination with antibiotics due to standard-of-care requirements (142, 172). Prior to the administration of phage to patients, preclinical workups can involve testing for synergistic effects of candidate phages with select antibiotics. Timing of antibiotic administration relative to phage administration also needs to be considered since phages require bacterial growth for replication. Antibiotics can slow or prevent bacterial growth, thus preventing phage self-propagation. In these cases, phage selection is based partly on the antibiotic susceptibility endpoint. Using a rat model of *P. aeruginosa* experimental endocarditis, one study showed that phage-antibiotic combinations were more effective at clearing the infection than phages alone (173). Interestingly, no phage-resistant mutants were recovered following *in vivo* administration, likely due to the reduced fitness and subsequent immune clearance of any resistant strains (173). In contrast, in a mouse model of systemic *P. aeruginosa* infection, resistant mutants were isolated from some anatomical sites following administration of phages alone (174). Notably, phage-resistant isolates were recovered from the lung. This demonstrates that differences in microenvironments and immune responses throughout the body can affect the efficacy of phage therapy. Careful analysis of such resistant mutants to identify specific mutations and using those mutants to iteratively select for additional phages that target newly exposed receptors will improve outcomes. The same study showed that phage cocktails were more efficient than single phages at reducing systemic bacterial load, and no phage-resistant isolates were recovered when antibiotics were co-administered (174). This suggests that *in vivo*, the use of phage cocktails and/or co-administration of phages with antibiotics is needed to increase treatment efficacy and prevent relapse.

Adapting (also known as training) phages by co-evolving candidate phages with target bacteria can increase the efficacy and specificity of phages. Using this method, phages were trained to more effectively clear bacteria and delay emergence of resistance in CF isolates *in vivo* (175, 176). Although there are limited examples of phage training related to treatment of *P. aeruginosa* infections, this strategy is often used in other species (177–179) and *in vivo* research. For example, phage PO4 was originally reported to only infect *P. aeruginosa* strain PAK (95). More recent work shows that PO4 can also infect strain PAO1, likely due to phage adaptation that allows recognition of a broader range of receptors (180).

### Clinical case studies of phage therapy for *P. aeruginosa*

Case reports of phage therapy for the treatment of refractory bacterial infections provide useful insights into the ways in which human immune responses and inter-patient differences might complicate phage therapy outcomes. The efflux system-targeting phage OMKO1 was combined with ceftazidime to successfully eradicate an antibiotic-resistant *P. aeruginosa* infection of an aortic graft (25). While the patient responded to previous administrations of ceftazidime, treatment with antibiotics alone did not clear the infection, but the addition of phage therapy was successful. In a similar post-surgical complications case, a phage cocktail and antibiotics (ceftazidime and colistin) were co-administered to clear a highly resistant *P. aeruginosa* infection of a prosthetic knee (181). In instances of implants or prosthetics where the bacteria are typically growing as biofilms, surgery and direct irrigation or application of the treatment to the site of infection can be required. This strategy ensures high localized titers of antimicrobials and phages, which are necessary to combat such difficult infections. Similarly, chronic urinary tract infections (UTI) benefit from direct instillation of phages into the bladder. In a case of UTI caused by antibiotic-resistant *P. aeruginosa*, the co-administration of a proprietary six-phage cocktail (Pyophage #051007) with meropenem and colistin led to eradication of infection (182).

In two separate cases of CF patients with antibiotic-resistant *P. aeruginosa*, administration of individualized phage cocktails containing LPS-targeting phages plus antibiotics successfully cleared the infections (183). The phage cocktails were administered intranasally, while antibiotics were administered intravenously. Bacterial isolates collected from both patients following phage administration were still pan-resistant after one week of treatment (the end point for patient one) (183). After two weeks of treatment, isolates from patient two showed mixed resistance profiles: some remained pan-resistant, while others became susceptible to beta-lactams (183). In another CF case, administration of a phage cocktail and antibiotic (ciprofloxacin and piperacillin-tazobactam or doripenem) cleared extensively resistant *P. aeruginosa* (184). Prior to phage administration, some isolates were sensitive only to the last-resort antibiotic colistin, which suggests that phage therapy may have helped to steer these strains toward antibiotic sensitivity. In contrast to *in vitro* studies of steering, the results were less consistent between patients, likely due to variability in immune responses and pathogen diversity, which affect steering outcomes.

Animals can also be afflicted with antibiotic-resistant *P. aeruginosa* infections. Diverse phages have been shown *in vitro* to infect *P. aeruginosa* isolates from various animals including, but not limited to, dogs, cattle, pigs, and cats (185, 186). A combination of phages and antibiotics was used to clear an implant-associated *P. aeruginosa* infection in a cat, showing successful treatment of biofilm-associated disease (187). Canine otitis externa is often associated with *P. aeruginosa* infections, and both multi- and single-phage applications have successfully treated antibiotic-refractory cases (188, 189). Similar to the problem for human case reports, the receptors for many therapeutic veterinary phages are often not identified or reported. Phage therapy in veterinary medicine is also a growing field, and there may be innovations in this area that precede those in human medicine due to the less stringent regulatory requirements for veterinary clinical trials compared to those for humans. Successful examples of phage therapy in veterinary medicine can guide innovation and new approaches in human health.

## CONCLUSIONS AND FUTURE PERSPECTIVES

### Current landscape of phage therapy

As successful examples of phage therapy continue to be reported, this modality is on track to solidify its status as a legitimate approach for treatment of antibiotic-resistant bacterial infections. Most countries administer phage therapy on a case-by-case/compassionate use basis, while global leaders such as Australia, Belgium, and Poland are paving the way for development of sustainable and safe centralized programs for phage therapy (160, 190–192). In a “top-down approach,” Phage Australia and Australian healthcare systems have developed three distinct pathways for compassionate use and clinical trials, as well as a clinical protocol (Standardized Treatment and Monitoring Protocol for Adults and Pediatric Patients, STAMP) to monitor phage therapy administration practices (160, 192). They focus on standardizing and coordinating phage therapy across the country and its integration into the broader healthcare system. A wider adoption of such programs in other countries can increase the integration of phage therapy as a useful treatment option.

### More emphasis on receptor identification is required.

Historically, therapeutic phages were not well characterized prior to administration. For example, IntestiPhage is a commercially available cocktail from the Eliava Institute in Georgia. It was first prepared by phage pioneer Felix d’Herelle in the 1920s and has since been continually adapted from the original formulation (193). Its exact contents have been undefined for over a century. This is clearly incompatible with modern regulatory standards for preparation of human-targeted therapeutics. Instead, Belgian regulatory agencies and researchers developed a detailed product monograph (first edition released in 2018) outlining standards for therapeutic phages (phage active pharmaceutical ingredients), where preparation methods, necessary phage information, and quality standards are described (161). Phage Australia also has a phage product quality control document with similar data requirements, as well as inclusion of *in vitro* phage susceptibility studies (190). Surprisingly, while phage genome and morphology are considered key pieces of information, phage receptors are not (161, 190, 194). Identification of receptors is important as it guides the design of phage cocktails to include phages that use different targets, reducing the rate of resistance. The phages in cocktail BFC1 are characterized (sequence, morphology, presence of virulence genes, host range). While BFC1 contains three phages, 14-1, PNM, and PT-7 (143), receptors for only the former two are reported (LPS and T4P, respectively) (195, 196). This pattern is also seen in single-phage administration. For example, *P. aeruginosa* phage PASA16 has been used therapeutically multiple times (197). The phage is well characterized, but its receptor is not specified (198). The authors provide a TEM image that can guide hypotheses regarding the target, but it remains unclear since there are many outer membrane candidates (198). Lack of receptor definition suggests that their identity was not considered in the selection of phages for therapy. Some phage receptors continue to be uncharacterized despite ongoing use of these phages (199). However, we acknowledge that where receptors are not reported, it may not necessarily mean they are unknown.

Despite clinical success in using phages despite unknown or unidentified receptors, there are still cases where patients do not respond to phage therapy. In the landmark 100-case observational study by Pirnay et al., treatment failure was often associated with chronic infections, especially in the lung. Bacteria from chronically infected patients may have altered expression of canonical receptors such as T4P and LPS due to lengthy interactions of bacteria and their associated phages in a confined environment. Phage-driven receptor loss renders new phages targeting the same receptor ineffective.

Identifying phage receptors has traditionally been of lower priority than sequencing phage genomes or examining capsid morphology by electron microscopy. Therefore, we suggest that systematic identification of phage receptors will allow for more targeted approaches, increase treatment efficacy, avoid trial and error, and save resources. We recognize that phage receptor identification is not always straightforward, since phages can use multiple receptors or receptors might be encoded by essential genes (which therefore cannot be deleted to evaluate the impact on phage infection) (57, 200). These situations will require new strategies to identify the receptors, including replacement with sequence-diverse homologs (15). Simply ruling out known receptors will already provide useful new information.

### The application of AI in phage receptor identification and therapy

The rapid rise of powerful artificial intelligence (AI) applications in the biological, chemical, and physical sciences has aided the structural characterization of proteins of unknown function and the development of novel peptides and compounds with therapeutic potential (201–203). In 2020, Belgian phage scientist Jean-Paul Pirnay shared a futuristic idea, set in 2035. He described a hypothetical small device that uses AI to produce a *de novo,* tailored therapeutic phage product for a selected bacterium (204). This concept sounds like science fiction, but the concept of using machine learning to aid phage therapy is already in development. Current AI technologies may be leveraged in phage therapy by predicting the target(s) of phage or even guiding *de novo* creation of receptor binding proteins that recognize a specified target. Similar efforts have been used to develop *de novo* peptides that bind to target proteins (205). This approach can broaden or change the receptor range of phages, potentially reducing resistance and increasing host ranges. AI can also help in the laborious process of defining phage susceptibility patterns for clinical isolates and guide the creation of phage cocktails. Using an in-house predictive model, Gaborieau et al. created phage cocktails that effectively killed *E. coli* clinical isolates (206). Since phages are so genetically diverse, it can be challenging to predict the type or variant of a particular receptor that a phage will recognize from sequence alone. Therefore, deliberate collection of receptor data will improve our fundamental understanding and allow us to train better AI models to make accurate matches between phage and host.

### Engineering and adapting phages can improve therapeutic candidates

In the future, phage engineering may play a larger role in phage therapy. Where natural phages for a particular isolate are hard to identify, genetic engineering or directed phage evolution could be used to create those that can recognize appropriate and/or available receptors. Swapping receptor-binding protein–encoding genes between related phages can confer the ability to bind new targets (15) or modify the host range of a phage if the new proteins can recognize a broader set of receptors. Mutating receptor binding proteins could also expand or change host range, allowing the phage to use a different version of the original receptor. As an example of this concept, *E. coli* phage λ, which uses the outer membrane protein LamB as its receptor, was evolved to recognize a new receptor (OmpF) for infection (177). While there was heterogeneity in the evolved λ phages, most of them still relied on the original receptor in addition to OmpF for productive infection (177). These studies suggest that evolving or engineering phages to recognize a completely new receptor may be more difficult than adapting them to recognize a homolog of an existing receptor. Engineering phages for recognition of new receptors is not yet a common approach in phage therapy, likely due to the few examples of successful phage engineering in human health, and the technical challenges of modifying phages that may have unusual codon usage or other genetic oddities (207). Because there is such natural diversity in phage receptors, the field has focused mainly on defining and exploiting existing receptor patterns. More research on this type of phage engineering and its applications is required for wider implementation in phage therapy.

In addition to receptor-based alterations, phages can also be engineered to display proteins. The ability of lysogenic phages to express modified versions of their bacterial receptor or enzymes that modify the host surface to prevent superinfection by related phages (32, 39, 65) could be co-opted to express surface-exposed factors that enhance phage or immune recognition and clearance of the bacterial host. This concept is similar to the strategy used in CAR-T cell therapy for cancer, where patient-specific T cells are engineered to express distinct antigen receptors that recognize relevant tumor-expressed factors (208). Another form of phage engineering involves using phages as vehicles for therapeutic cargo. In one example, *E. coli-*specific phages were engineered to carry multiple CRISPR-Cas systems that targeted *E. coli* genomic DNA, resulting in Cas-mediated cell death (209). These CRISPR-Cas–armed phages (CAPs) self-amplified at a higher rate than ancestral phages, outperforming typical phages in key therapeutic qualities (209). A similar phage product in development targets *K. pneumoniae* strains that cause urinary tract infections (210, 211). This engineered phage also encodes a DNA-targeting CRISPR-Cas system but maintains the phage’s lytic activity, providing two complementary bactericidal strategies (211).

Phage therapy has a bright future, as experts from many fields are interested in harnessing the power of these viruses. We suggest that more careful consideration and better understanding of receptor profiles are important facets of future work. As surface-exposed structures at the interface between bacteria and their environment, phage receptors play multiple roles. Phage steering directs the loss of these key bacterial components, and their identity should be key drivers of phage cocktail design. Identification of new phages that use non-canonical receptors can contribute to more robust therapeutic outcomes. These considerations will also guide the development and expansion of phage libraries and biobanks as resources for clinicians, reducing the time between diagnosis and treatment. More creative approaches to phage isolation are needed, as the host strains and growth conditions we use impact which phages we find; changing our search strategies to include new sources of phages and screening under more patient-relevant growth conditions will diversify our discoveries. Normalization of phage receptor identification and reporting will increase the success of phage steering.
